# Climate change in the Catalan Pyrenees intersects with socioeconomic factors to shape crop diversity and management

**DOI:** 10.1007/s13593-022-00806-3

**Published:** 2022-09-02

**Authors:** Joana Blanch-Ramirez, Laura Calvet-Mir, Laura Aceituno-Mata, Petra Benyei

**Affiliations:** 1 Institut de Ciència i Tecnologia Ambientals, Universitat Autònoma de Barcelona, Building Z Campus UAB, 08193 Barcelona, Bellaterra (Cerdanyola) Spain; 2 Internet Interdisciplinary Institute (IN3), Universitat Oberta de Catalunya, Av. Carl Friedrich Gauss, 5. Parc Mediterrani de la Tecnologia, Castelldefels, 08860 Barcelona, Spain; 3 Instituto Madrileño de Investigación y Desarrollo Rural, Agrario y Alimentario (IMIDRA), Finca El Encin, Autovía del Noreste A-2 Km 38,2., 28805 Alcalá de Henares, Spain

**Keywords:** Adaptation, Agricultural industrialization, Agrobiodiversity, Home gardens, Landraces, Traditional ecological knowledge

## Abstract

Most studies on climate change’s impacts on agriculture focus on modeling techniques based on large-scale meteorological data, while few have investigated how farmer’s perception of climate change’s impacts can affect crop diversity and crop management practices, especially in industrialized contexts. To fill this gap, we conducted 24 semi-structured interviews in a study site located in the Catalan Pyrenees. Our results show for the first time in an industrialized context that farmers perceive multiple interrelated climate change impacts on local agroecosystems. For instance, snowfall and freeze events have decreased, which respondents associated with the increase of pests and diseases affecting both wild flora and cultivated plants. Similarly, changes in precipitation patterns lead to a perceived decrease in useful rain for agriculture. Farmers are also reporting changes in their management practices, such as increased irrigation or use of pesticides, which respond to these climatic factors but also to changes in the crops that are cultivated. Crop diversity is in decline in the area both at the species and landrace levels, especially in rainfed fields. This is mainly driven by socioeconomic factors such as agricultural abandonment or access to commercial seeds, although climate change factors such as increased pests or decreased rainfall can have an impact. Despite the crop diversity losses found, many landraces have been maintained, mainly due to their cultural value, and also new crop species have been introduced, which are now viable due to the increase in temperature. Although we focused on a specific case study, we found several trends that are also present in other contexts. Therefore, the results of this research are relevant at a global scale since they show that climate change is affecting mountain agroecosystems in industrialized contexts and may affect more drastically both agrobiodiversity and crop management practices in agroecosystems worldwide.

**Supplementary Information:**

The online version contains supplementary material available at 10.1007/s13593-022-00806-3.

## Introduction

Crop diversity, or the variance in genetic and phenotypic characteristics of plants used in agriculture, is the result of millennia of favorable trait selection, aiming at producing larger, earlier ripening, culturally appropriate, or frost-resistant crops (Goland and Bauer 2004). Breeding lines, wild progenitors, related species, and landraces (also called local varieties, traditional varieties, heirloom varieties, or farmer varieties) are part of this diversity (Negri 2005; Riu-Bosoms et al. 2014). Specifically, landraces have been defined as dynamic populations of a cultivated plant with a historical origin, distinct identity, often genetically diverse and locally adapted, and associated with traditional farming systems (Camacho Villa et al. 2005). Indeed, landraces have been evolving in association with traditional knowledge and local culture over time, continuously changing as the environmental and socioeconomic conditions changed (Negri 2005).

Generally speaking, crop diversity contributes to the resilience of farming systems, while crop homogeneity makes farming systems more vulnerable to ecological, political, and economic disturbances (Wolff 2004; Rotz and Fraser 2015). Indeed, some studies have pointed out that agrobiodiversity can be a robust way towards sustainable development (Altieri and Nicholls 2017) and crop diversity has a prospective important role to play in crop adaptation to climate change (de Carvalho et al. 2016). For example, landraces could be crucial to maintain healthy agroecosystems since crop genetic uniformity is directly associated with pest invasions and outbreaks (Altieri et al. 2015). Moreover, landraces can improve soil quality, increase carbon sequestration in the soil, reduce the need for agrochemicals, and promote the recovery of associated wild biodiversity (Carranza-Gallego et al. 2018). This is why several policy agreements, such as the Convention of Biological Diversity (CBD), the Agenda 21, and the International Treaty on Plant Genetic Resources for Food and Agriculture (ITPGRFA), have emphasized the need to promote crop genetic diversity conservation in the form of landrace conservation programs as a component of sustainable agriculture (Veteläinen et al. 2009).

And yet, paradoxically, there are several reports of global landrace abandonment trends (Negri 2005; Martin et al. 2019; McLean-Rodríguez et al. 2019). Historically, humans have used more than 7000 plant species for food and agriculture, but only 150 species are under extensive cultivation nowadays (Thrupp 2000; Gepts 2006), and only about 100 species are used in 90% of the world’s food cropping systems (FAO 1997). Indeed, according to FAO (FAO 1999, 2004), about 75% of the genetic diversity found in agricultural cropping systems has been lost over the last century.

In order to revert this genetic erosion, it is important to understand crop diversity trends through time and the global and regional socioeconomic and climatic events that drive those trends. Even if the causes of agrobiodiversity loss are multiple and interrelated, those most reported by the existing literature refer to socioeconomic and historical factors. These include agricultural industrialization and commoditization, rural de-population, low prices of agricultural products, and technological changes that have led to the spread of monocultures where subsidized, low-labor demanding crops and modern hybrid varieties predominate (Naredo 2004; Wolff 2004; Negri 2005; Cebolla-Cornejo et al. 2007; Aceituno-Mata 2010; Calvet-Mir et al. 2011; Riu-Bosoms et al. 2014; Sardaro et al. 2016). However, there is also abundant literature about climate change impacts on agroecosystems. This includes reports on how climate change will affect crop productivity due to the increased climate variability, and the changes in seasonal, precipitation, and temperature patters that will affect crop growth and crop pests and diseases (Dangi et al. 2018; Aguirre-Liguori et al. 2019; Birhanu Abegaz and Hailu Tessema 2021). Still, there is a predominant focus on using modeling techniques based on data from experimental plots, temperature and precipitations records, or remote sensing (Mann and Gleick 2015), and studies looking specifically at the impacts of climate change on crop diversity as perceived by farmers themselves are not that abundant. Indeed, some authors have highlighted the relatively untapped potential of looking into how the local perception that farmers have about climate change impacts on agroecosystems can shape crop diversity and management choices (Soubry et al. 2020; Labeyrie et al. 2021). This data source remains largely unexplored in industrialized contexts such as the European one.

**Fig. 1:**
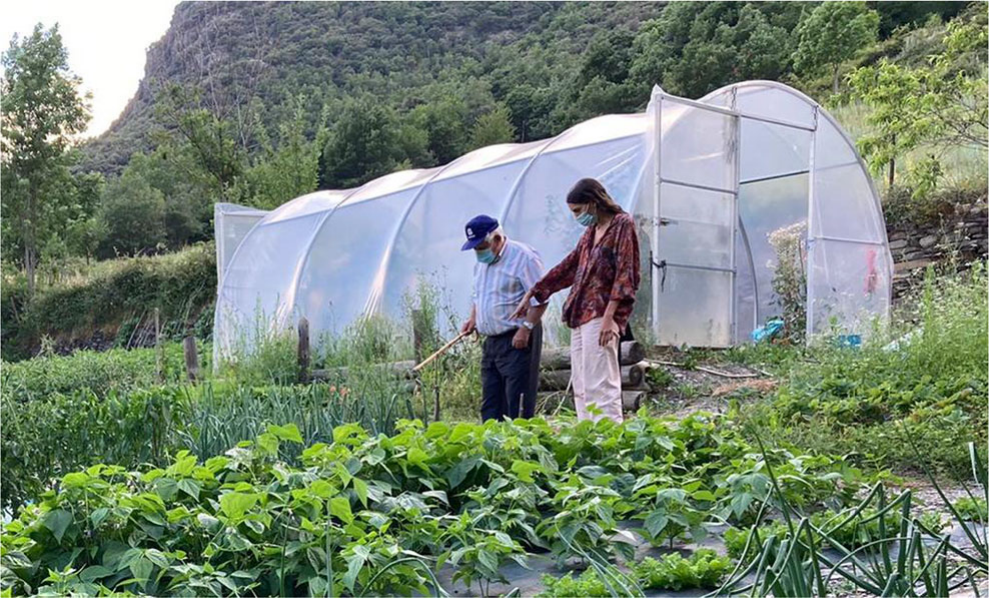
A farmer from the study site explains the different crops he grows. Credit: Joana Blanch.

To fill this gap, we conducted fieldwork (interviews and participant observation) in a study site in the Catalan Pyrenees. The site was selected because of its relative isolation (leading to better-preserved traditional knowledge systems) and the fact that farmer communities there have had long-term interactions with the environment. Our main objectives were (1) assess the perceived climate change impacts in the study area, (2) explore how farmers have changed their crop management practices to adapt to those impacts, (3) document changes in crop species and landraces, and (4) identify the climatic and socioeconomic factors driving those crop changes. To narrow down the scope of the study, we focused on the home garden agroecosystem when asking about changes in crop management and landraces (see Fig. 1).

## Materials and methods

For this research, we adapted the LICCI data collection protocols (see Labeyrie et al. 2020; Reyes-García et al. 2020 for more details) that are specifically constructed to explore the local indicators of climate change impacts perceived by local communities and the crop diversity trends based on local knowledge (see Supplementary materials for more details on our adaptation of the original methods). This study is part of a larger project on local indicators of climate change impacts on crop diversity that is taking place in five mountain agroecosystems of the Iberian Peninsula (Cabrales, Alpujarra Alta, Muntanyes de Prades, Vall d’Àssua, and Vall de Cardós). Qualitative data was collected using semi-structured interviews and participant observation in Vall de Cardós, northern Catalonia, between July and August 2020. This project had received the ethical approval of the Ethics Committee of UAB (CEEAH 4781 and CEEAH 5108).

### Study site

**Fig. 2:**
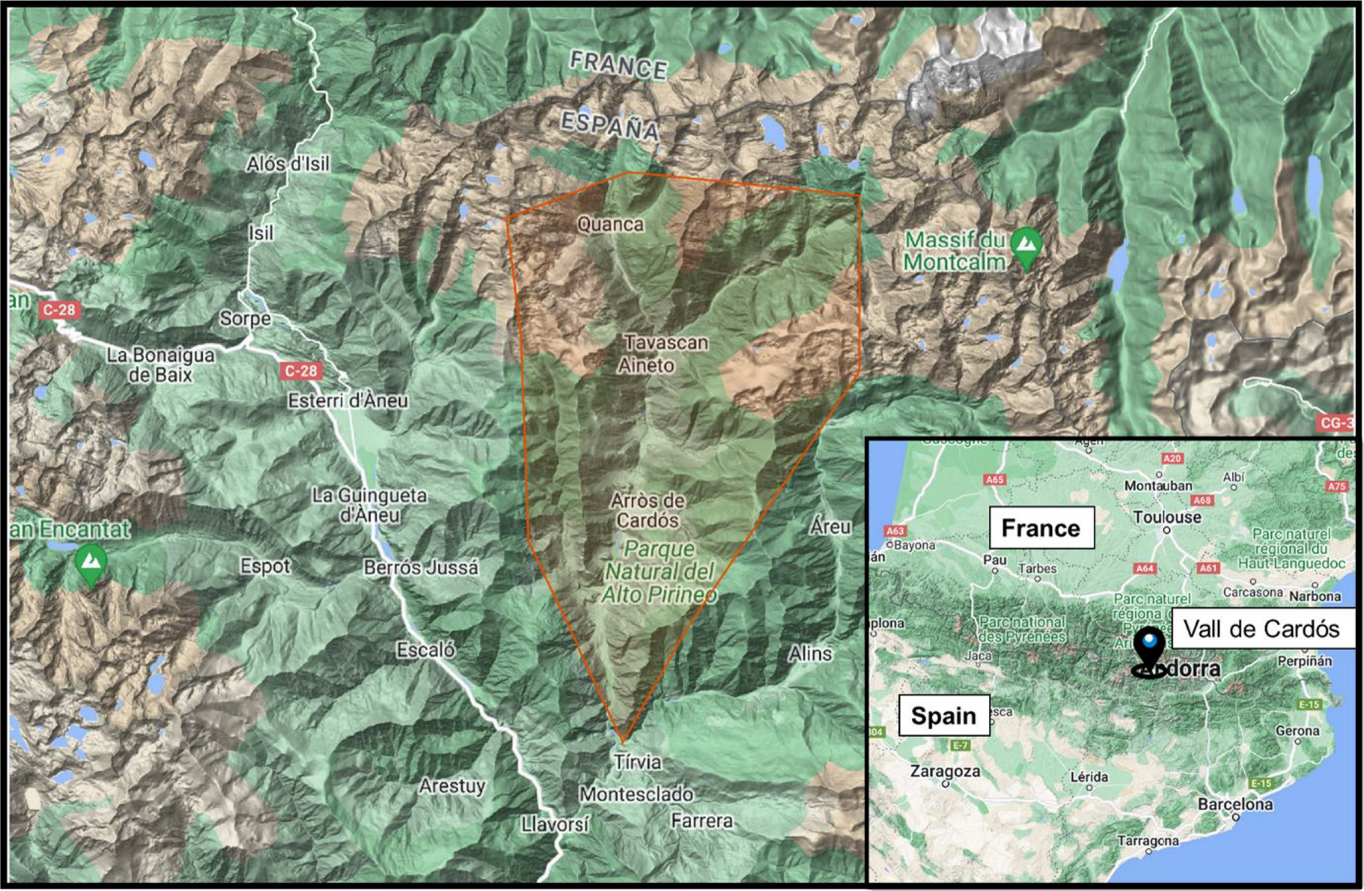
Regional map of Cardós Valley. Source: Google My Maps.

Vall de Cardós is a glacial Pyrenean valley situated in the north of Pallars Sobirà county (Fig. 2). It includes the municipalities of Esterri de Cardós, Lladorre, and Vall de Cardós with a total area of 220 km^2^ (Idescat 2019). The total population is 664 inhabitants, and the valley has had a demographic evolution similar to others Pyrenean valleys, where population declined considerably during the second half of the twentieth century, although during the last decades there has been a certain recovery due to tourism. Part of the study site is included in the Natural Park of High Pyrenees, and the river Noguera de Cardós is the backbone of the area. The altitude in the region varies from 898 masl at the bottom of the valley to nearly 3000 masl at the peaks of the mountains. The orography factor determines the climate of Vall de Cardós: summers are cool, and winters are long and cold. There is a significant climatic contrast between the different seasons of the year, and the average annual temperature is 10°C (Sudrià i Andreu 2003).

Normally, the rainiest season of the year in the Pyrenees is summer; however, Vall de Cardós is the great exception of the Pyrenean territory. In this valley, the wettest season is spring, followed by autumn (de Gràcia 2016). The annual precipitation has an altitudinal gradient, and it varies from 700 to 1100 mm. Also, snow is a frequent phenomenon during the winter.

The climatic staggering, together with the complicated orography, determines the type of vegetation and consequently the human activity. Four vegetation layers can be differentiated: snow line, alpine, subalpine, and montane zone. The snow line zone is located around 3000 masl, and it has a rocky appearance due to frosts. The alpine zone is situated between 2300 and 3000 masl, and it is the realm of pasture, with some mountain pine (*Pinus uncinata*). The subalpine zone is between 1600 and 2300 masl and is dominated by coniferous forests, mostly mountain pine and firs (*Abies sp.*). It is the most sensitive habitat to human action as it has been intensively exploited to supply coal and wood. The montane zone occupies the bottoms of the valleys, and it is composed of deciduous forests and riparian trees that follow the river’s courses such as oak, ash, birch, poplar, alder, or willow. Human presence has altered these ecosystems converting them into mowed meadows to feed livestock. However, the gradual abandonment of traditional livestock has recently triggered the expansion of forest cover (Sudrià i Andreu 2003).

The isolation of this valley until relatively modern times (the road was first opened in the 1930s and completed in the 1950s) contributed to maintaining an agrarian economy based on self-sufficiency (Mateu i Llevadot 1983). In the mid-nineteenth century, wheat, rye, barley, potato, some fruit trees, and grass were grown. Cattle of different kinds were raised, but especially cattle and mules for fieldwork. Other common activities were partridge hunting and trout and eel fishing. Recently, the increase in tourism has been replacing the traditional primary sector of the Vall de Cardós. However, tourism activities are concentrated only in summer, especially in August, and the benefit they bring to the valley’s population are scarce (Sudrià i Andreu 2003).

### Data collection

Our research was conducted in seven villages selected from a total of 17 villages geographically located in the Vall de Cardós that had similar altitude and environmental conditions. The selection of the villages was carried out to have a representation of the three municipalities of the valley and of the environmental and socio-cultural conditions of the site. Villages without home gardens or permanent residents were excluded.

Data was collected via 24 in-depth semi-structured interviews (SSI), participant observation (the first author lived in the valley during the research period), and a field diary to understand the context of the site, the perceived local indicators of climate change impacts (LICCI), the changes in crops, landraces, and management practices over time and the drivers and consequences of these changes.

Interviewees were selected based on purposeful and snowball sampling, targeting elder farmers (60+ years old) with long-term experience and close relationships with (a) the local environment in general and/or (b) the home garden agroecosystem. Specifically, 22 home gardeners (17 women and 5 men) were interviewed (managing a home garden for at least 25 years), and two elders that, despite not having a home garden, had sufficient long-term knowledge about the local environment to be able to perceive climate change impacts and general changes in cropping systems. The sample’s gender balance is biased towards women because, in this area, women have historically been in charge of home gardens and seed management.

Interviews (that lasted about one hour each) were digitally audio-recorded after obtaining agreement from participants (free prior informed consent forms and oral permissions). Interviews were conducted directly in the home gardens or at the interviewee’s houses but always following the LICCI project safety and security measures adapted to the COVID-19 pandemic. The interviews were performed following a script with different sets of questions, and they were conducted in Catalan (see Supplementary materials for more detail). This script was based on the data collection protocols of the LICCI project and included questions such as “Have you perceived any change in the local environment (e.g., rain, soil, water, plants animals)? And in the cropping systems in the area? If so, what is driving this change? And have you done anything to adapt to those changes?”.

To analyze changes on a temporal scale, the interview questions included a relationship to the past, referring to when the interviewee was young and to specific historical events that were known (e.g., when the road was built). In cases where the interviewee was clear about the time of the observation, he or she was asked specifically about the year in which the change occurred. Moreover, a catalogue of landraces (Arribas Quintana et al. 2011) was shown to the respondents to contrast their information and to identify possible synonyms or homonyms of landraces in cases where the identification of landraces was unclear. This catalogue was produced by the County Council in 2011 and is based on a set of descriptive sheets of 23 landraces of the Pallars Sobirà region, accompanied by photographs.

### Data processing and analysis

After fieldwork was conducted, notes from the interviews and audio recordings were revised, and a series of tables were completed with the aggregated information from all the interviews. Four tables were created and obtained regarding climate change impacts, crop management practices, and crop diversity changes at both species and landrace levels. These tables were later analyzed using descriptive statistics to obtain the number of mentions and the relative percentage of those mentions.

The observations of climate change impacts were categorized and clustered according to the classification of the LICCI protocol (Reyes-García et al. 2020). This classification is based on four systems (climatic, physical, biological, and socioeconomic system), which in turn include different subsystems (e.g., temperature, precipitation, air masses, and seasons for the climatic subsystem) that break down into different impacted elements (e.g., mean temperature, temperature extremes, seasonal temperature, and sunshine for the temperature subsystem). For each impacted element, there are several defined and specified LICCI categories that have been used to classify and quantify the observations and mentions.

Concerning crop diversity changes, the information given by the interviewees was clustered to obtain lists of crop species and landraces that were cultivated in the past and their current permanence or abandonment, as well as of crop species and landraces that have been introduced and were not grown in the past. The identification of landraces was done following the nomenclature used in the Spanish Inventory of Traditional Agrobiodiversity Knowledge (Tardío et al. 2018). To solve problems of identification, we consulted the book “30 varietats tradicionals de l’Alt Pirineu” (Col·lectiu Eixarcolant 2020) published in December 2020, and we also contrasted our information with Marc Talavera, one of the book authors.

In addition, the audio recordings were revised to identify the climatic and socioeconomic factors driving changes in crop species and landraces. With this information, we created a mental map with Miro software that synthesizes the information about the interconnected links between the reported changes and the driving socioeconomic and climatic factors.

Finally, we calculated the number of mentions for each change and the number of respondents who have observed the same change. Percentages were calculated from the total number of mentions (108) and the total number of respondents (24).

After data processing and analysis, we conducted five follow-up interviews with people from the same sample to clarify questions and resolve contradictory information.

## Results

### Perceived local indicators of climate change impacts on Vall de Cardós’ agroecosystems

All of the interviewees indicated at least one perceived change in the climatic, physical, biological, or socioeconomic systems over time that were directly or indirectly impacting the local agroecosystems, primarily the home gardens (see Table 1). Applying the LICCI classification system to our observations resulted in 32 local indicators of climate change impacts categorized in seven subsystems and four systems (Table 1).

**Table 1:** Local indicators of climate change impacts (LICCI). Frequency is proportional to the number of mentions.

Subsystem	Observation	Mentions
Climate		
Precipitation	Nowadays it rains less than in the past (mean rainfall)	7
	Nowadays rains are heavier than in the past (changes in the intensity of rainfall)	1
	Rain is now more variable (changes in variability of rainfall)	4
	Now it is harder to predict when it is going to rain (changes in the predictability)	2
	Now there is more drought than before (frequency of drought events)	3
	Before it rained more often (temporal distribution)	1
	Now there is less rain in summer (changes in the amount of rainfall in a given season)	3
	Now there is less rain in spring (changes in the amount of rainfall in a given season)	2
	**Total**	**23 (21.3%)**
Temperature	Nowadays it is warmer than in the past (increase of the mean temperature)	8
	Increase in the frequency of cold waves	1
	Now the temperature is more variable than before	1
	Now the frequency of days with extreme temperatures have increased	3
	Now summers are warmer (changes in the mean temperature in a given season)	4
	Now it is less cold in winter (changes in the mean temperature in a given season)	5
	Now there are more unusual temperatures in a given season (frequency)	1
	**Total**	**23 (21.3%)**
Seasons	Before seasons were more defined (changes in the transition between seasons)	**8 (7.4%)**
Cryosphere (ice and snow)	Nowadays it snows less than in the past (changes in the amount of snowfall)	15
	Now it does not freeze as much as before (frequency of freeze events)	3
	Before the snows started earlier (changes in the calendar/temporal distribution of snowfall)	3
	Now the formation of snow cover has changed (temporary snow cover)	2
	Before, the snow cover used to last longer (duration)	1
	Now there are more late frosts (frequency of freeze events)	1
	**Total**	**25 (23.1%)**
Biological		
Terrestrial wild fauna	Now, there are many insects that die (mortality)	2
	Before, there were more bees (abundance)	1
	**Total**	**3 (2.8%)**
Terrestrial wild flora	There are fewer nuts now than before (productivity)	3
	Now the trees get more pests than before (occurrence of diseases/pests in wild flora)	2
	There used to be more walnut trees (abundance)	1
	**Total**	**6 (5.6%)**
Socioeconomic		
Agriculture (cultivated plant spp.)	Now there are more crop diseases and pests	13
	Now there is the potato beetle (crop pests)	2
	Now, some vegetables bloom later (changes in flowering time)	1
	Now there are more spider mites that damage the home gardens (crop pests)	2
	Now there are many birds in the orchards and fruit trees (crop pests)	2
	**Total**	**20 (18.5%)**

Most observations were reporting impacts in the climatic system (50% of the total mentions), although respondents also reported changes in the physical, biological, and socioeconomic systems (see Table 1 for details). In fact, most mentioned changes were related to changes in the cryosphere (23.1%), precipitation (21.3%), temperature (21.3%), and agriculture subsystems (18.5%).

The most affected subsystem was the cryosphere (ice and snow), and the most mentioned change pointed to the decrease in the amount of snowfall (15 respondents). Changes in the frequency of freeze events and the duration and temporal distribution of snow cover were also reported. An elder home gardener from Lladrós town stated, “It used to snow much more than it does now. Grandparents celebrated the snow in November and December because it meant that in summer the water from the springs would come down. Now it snows less and there is less water in general”.

More than half of the respondents mentioned changes related to rainfall. The most reported change in this subsystem was the fact that it used to rain more than now (12 respondents), both at the average general rainfall level and in a particular season (in spring and summer). Moreover, six respondents said that now the rain is more variable or harder to predict. Nine interviewees also mentioned that this has a negative consequence for the yield of crop fields and home gardens. An elder farmer from Lladorre stated, “It may fall the same amount of water as before, but nowadays there are strong storms, and the water is not as useful as in the past. Before, rainfalls were more regular and predictable; the rain was light so that the water could soak into the soil”. Reports of temperature changes (13 respondents) were also abundant and were mostly related to mean temperature increase (eight respondents) and an increase in temperature extremes (four respondents).

Another much-mentioned observation was the change in the transition between seasons. Eight interviewees observed that in the past, seasons were more defined and transitioned more gradually than now.

Regarding the terrestrial wild fauna subsystem, two respondents stated that now the mortality of insects has increased, and one interviewee mentioned that bees before were more abundant. Respondents associated this imbalance in insect populations (decrease of beneficial insects such as bees and increase of pests) with changes in the terrestrial wild flora subsystem. Specifically, respondents stated that trees abundance and productivity have decreased, while mortality has increased. The decrease in the abundance of insects may be also due to an increase in the use of insecticides according to home gardeners.

Despite this general trend of decrease in the abundance of insects, there is a perceived increase in pests affecting both wild trees and cultivated plants. Local perceptions associate this trend with climate change, as a home gardener from Cassibrós stated “Now there are more pests than before because in winter is not as cold and it snows less; therefore pests can survive easier”. Indeed, the most mentioned impact in the socioeconomic system, specifically to the agriculture subsystem, is the increase in crop diseases and pests, mentioned by 13 respondents. Home gardeners reported two major drivers of this increase. On the one hand, as snowfall and freeze events have decreased and temperatures have risen, pest populations have grown faster. Crop diseases follow a similar pattern particularly in the case of pathogens like fungi. Therefore, the spread of crop diseases and pests is linked indirectly with climate changes. On the other hand, interviewees also believe that this increase in pests and diseases is due to the decrease of beneficial insects for pest control such as ladybugs. For example, a respondent from Ribera de Cardós stated, “There used to be more ladybugs, bees, earthworms…These animals are good for home gardens since they prevent certain pests such as aphids”. Moreover, two respondents also mentioned that now there are many birds in the home gardens and fruit trees. The respondents’ explanation is based on the fact that there are no grain fields now where the birds could supply their feeding needs and therefore birds search now for additional food in home gardens. Finally, another perceived change in the agriculture subsystem includes changes in crop flowering time, which respondents relate to changes in precipitation and temperature patterns.

### Adaptations to climate change impacts through home garden management changes

There has been a notorious change in cropping systems and management practices in the valley. The landscape has changed drastically as a result of crop abandonment and depopulation as well as due to socioeconomic transformation. In the early 1960s, the change of self-subsistence economy to a market economy began resulting in a rural exodus from the valley and a consequently gradual increase of the forest mass.

In parallel with this, respondents mentioned seven changes in management practices in home gardens directly or indirectly related to climate change (see Table 2 for more detail). A total of 20 interviewees reported a change in management practices, and half of them mentioned that nowadays, home gardens need to be irrigated more than in the past. According to the interviewees, this adaptation responds to the lower precipitation and the higher variability of rainfall patterns (see Table 1). For example, an elder from Benante town said “Before it rained more than now, no more than eight days went by without raining; so now home gardens need to be more irrigated”.

**Table 2:** Changes in management practices (i.e., adaptations) related to climate change and the local indicators of climate change impacts (LICCI) associated subsystem with the number of respondents who mentioned each adaptation.

Adaptation	Mentions	Climatic relation	LICCI associated subsystem
“Nowadays we water or we need to water the home gardens more than before”	10	Yes	Precipitation and temperature
“Now we use fewer pesticides than 30–40 years ago”	5	Indirectly	Diseases and pests (agriculture and wild fauna)
“Now we need to use more pesticides than in 50 years ago since there were fewer pests in the past”	11	Indirectly	Diseases and pests (agriculture and wild fauna)
“Now we sow or plant later than before”	6	Yes	Ice and snow and seasons
“Before potatoes, peas, beans, and cereals were grown in rainfed land and now it would not be possible”	8	Yes	Precipitation and temperature
“Today, there is a longer harvesting period in summer crops”	2	Yes	Precipitation and temperature
“New summer crop species are viable due to warmer and longer summers”	3	Yes	Precipitation and temperature

Eleven respondents have stated that they now need to use more pesticides than before, and this is related to the reported increase in pests (see Table 1). Indirectly, this adaptation is linked with the snowfall change perceived by the respondents as often the driver of this pests’ increase was mentioned to be the decrease in snow during winter. However, five respondents stated that about 40 years ago they used more pesticides than today. In the 1970s and 1980s, new pests appeared, and people started using pesticides. At present, pests have increased, but also the awareness about the impacts of pesticide overuse. Indeed, three interviewees said that today farmers are more aware of the negative impact of pesticides and they try not to use them as often as before. A home gardener from Ribera de Cardós stated, “People used pesticides a lot, now there is more awareness about health and the environment, so people try not to use so many chemicals as before”. Thus, although pesticides are more needed now due to the increase in pests, some farmers try to use them less because of environmental concerns.

Another adaptation related to climate change is the change in sowing and planting periods. Six respondents reported that nowadays they cannot sow and plant as early as before because now there are more late frosts that can damage their home gardens. For instance, an interviewee said, “We used to plant in early April and harvest earlier, now in April it can still freeze so it’s not worth planting until May 15th”. Therefore, this change in management practices is related to changes in the cryosphere and seasons’ subsystems (see Table 1). Moreover, eight farmers reported that before, they used to plant potatoes, peas, beans, and cereals using a rainfed system, but that today, it would not be possible due to the decrease in rainfall. As an elder from Lladorre stated, “Before, potatoes, beans, peas, and chickpeas were grown in the fields without irrigation; now it could not be done because it used to rain more regularly and now the weather is drier”.

### Changes and drivers of crop species and landraces diversity over time

**Fig. 3:**
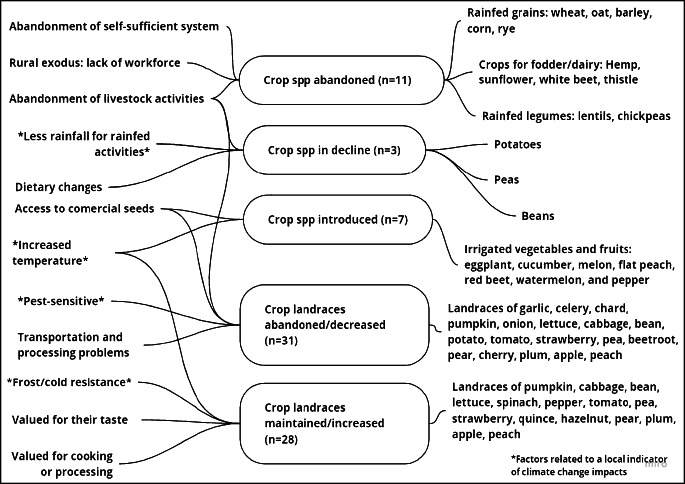
Mental map of changes and drivers of crop diversity in Vall de Cardós.

Respondents reported several changes in crop species diversity (see Table S3 in Supplementary materials and Fig. 3 below for details on the drivers of diversity). As mentioned before, the traditional economy of self-sufficiency and the isolation of Vall de Cardós implied the need to grow cereals and other crops both for human consumption and for livestock. In the past, wheat, hemp, chickpea, oat, sunflower, lentil, barley, maize, thistle, white beet, and rye were grown in rainfed fields. Nowadays, no one cultivates these crop species in the fields for different reasons. Therefore, eleven crop species have been abandoned.

Cereals (wheat, oat, barley, and maize), which in the past occupied large extensions of the valley, are no longer cultivated because nowadays, it is more profitable to buy the grain than to grow it. This is ultimately due to both the easier accessibility to the market and to the complicated orography, which makes the mechanization of agriculture in this valley difficult. Similarly, farmers used hemp for spinning fabrics and gave it to chickens and chicks. However, farmers no longer conduct these practices.

Moreover, some crop species such as sunflower, thistle, white beet, and potato have been abandoned because their use was related to specific livestock that has been lost. For example, sunflowers were given as fodder to dairy cows, which was a traditional agrarian activity in this valley that was intensified from 1932 onwards, when the first dairy cooperative was created. Nowadays, milk production is not profitable in the valley because feed for cows, transportation, and labor costs have increased. Therefore, most cows are beef cattle today, thus, no protein-rich fodder crops are required since cows are left in the pasture lands. In line with this, thistle was used as rennet for cheese production, but now, it is no longer produced in the valley. In addition, white beets were used for pigs’ consumption, but pig farming has been totally abandoned.

As explained so far, the abandonment of crop species is mostly due to socioeconomic changes. However, respondents also mentioned a potential climatic driver, the decreased rainfall, and the shift from rainfed cropping systems (fava bean, pea, and potato) to irrigated home gardens. As mentioned before, eight farmers stated that now it would be impossible to grow these crop species without irrigating them, and thus, decreased rainfall might be a potential underlying factor encouraging their abandonment.

In contrast with this crop species loss, there are seven vegetables and fruits that have been introduced, specifically in the home gardens: eggplant, cucumber, melon, flat peach, red beet, watermelon, and pepper. These species have been introduced due to dietary changes and access to commercial seeds and seedlings. Apart from these socioeconomic drivers, three respondents stated that the reason for the introduction of these species is also climatic changes that allow farmers to plant some species that would not have grown in the past (see Fig. 2). One of them said, “Now I have a flat peach tree that before would not have grown because now it is warmer”. The longer summer season allows the cultivation of cold-sensitive species that require warmer summers.

As per the landrace diversity, a list of 59 landraces cultivated by more than one generation has been obtained from the fieldwork (see Table S4 in Supplementary materials) along with the changes associated with the use of these landraces, the drivers of those changes, and the influence of climate change impacts.

Respondents mentioned eight landraces that are no longer grown and have therefore been lost. Moreover, many of the landraces still present in the home gardens are declining as the number of farmers who grow them is much lower than in the past. In fact, 25 landraces (41% of the total) have suffered a decrease in their use in home gardens of Vall de Cardós, leading to a loss of crop diversity. However, almost half of the landraces mentioned (45.9%) do not present any change or have increased their presence in home gardens.

The loss of landraces is due to several reasons. First, now people buy commercial seeds because it involves less labor work while in the past home gardeners sowed all the seeds themselves. For instance, an interviewee from Lladrós stated “Before, everything was seeded at home and now people buy seeds and plants because it is easier”. Therefore, one important driver of landraces’ changes is greater market accessibility. Moreover, some landraces have fallen into disuse because their seeds are not for sale, so people cannot access them unless they have someone that gives them seeds or they save their own seeds from one season to the next one. This is the case of *tomàquet del país* (tomato landrace). Other reasons are problems of transportation (as in the case of *préssec blanc* peach tree landrace), or processing difficulties (as in the *patata del bufet* potato landrace, which is difficult to peel).

The loss of landraces is due to several reasons. First, now people buy commercial seeds because it involves less labor work while in the past home gardeners sowed all the seeds themselves. For instance, an interviewee from Lladrós stated “Before, everything was seeded at home and now people buy seeds and plants because it is easier”*.* Therefore, one important driver of landraces’ changes is greater market accessibility. Moreover, some landraces have fallen into disuse because their seeds are not for sale, so people cannot access them unless they have someone that gives them seeds or they save their own seeds from one season to the next one. This is the case of *tomàquet del país* (tomato landrace). Other reasons are problems of transportation (as in the case of *préssec blanc* peach tree landrace), or processing difficulties (as in the *patata del bufet* potato landrace, which is difficult to peel).

Second, and related to the first point, some landraces are very pest sensitive, and therefore, they are not effectively adapted to the new climatic context with pests increase. As an example, a woman from Lladorre said that “Here, there used to be a giant perera de Cardós (pear tree) that always produced a lot of pears, but now this type of pear tree is disappearing due to pests”. Since there are not as many inhabitants as in the past, certain crop species and trees are not cared for, and these landraces are gradually being lost. A home gardener from Ribera de Cardós stated that “Nowadays, as fewer people are living in the area, fava beans are sowed less and are being lost”.

Despite this loss of crop diversity, there is still an important maintenance of landraces in home gardens (see Table S4 in Supplementary materials). This maintenance is due to organoleptic properties since home gardeners have a preference for certain tastes and smells associated with landraces that commercial varieties lack. Another important reason for this maintenance is the adaptation of landraces to the local climate, specifically to cold and snow. For example, *bleda de sempre* (chard landrace), *col de tocino* (cabbage landrace), or *enciam negre* (lettuce landrace) are landraces that respondents value because they are kept throughout the winter, they are cold and snow resistant, and so they do not freeze during the winter. However, due to warmer conditions, some landraces are not as valued as before since now they have a shorter harvest period, such as *enciam negre* (lettuce landrace), which spikes more rapidly than other commercial varieties.

Other reasons are the cultural and traditional appreciation of certain landraces and the maintenance of dishes and culinary preparations that involve these landraces. Also, the diversity of landraces allows some crop species to be present throughout the year; this is the case with cabbage. Home gardeners have different landraces of cabbage that are harvested in different seasons, and in doing so, they can eat cabbage all year round. Similarly, some landraces are valued for their long conservation, such as *ceba valenciana* (onion landrace), *pomera de Cardós* (apple tree landrace), and *pomera morro de llebre* (apple tree landrace).

The last reason that respondents mentioned related to the importance of maintaining landraces is the fact that traditional seeds sometimes give more productive crops than commercially grown and bought plants. This depends on the type of crop species and landrace. For example, two farmers complained that when they buy a potato plant, it is not as productive as when they plant their own landrace. Others also mentioned that when they buy a plant, it already has pests or diseases (such as the potato beetle), so they prefer to sow themselves the landraces and avoid buying the commercial variety.

## Discussion

In this section, we discuss our results focusing on (1) the perceived impact of climate changes on home gardens and changes in management practices to adapt to those impacts, (2) the changes in crop species and landraces, and (3) the climatic and socioeconomic factors driving those changes. Before all else, we account for a set of caveats to interpret the results correctly.

### Caveats

First, the list of landraces stems from respondents’ individual reports, without the possibility of conducting focus group discussions to get a cultural consensus agreement about landraces due to the COVID-19 sanitary situation. Therefore, there may be a repetition of the same landrace since it could be named differently in different towns or households. Similarly, two different landraces may have the same name, but different genotypes. To solve this limitation, we used a previously published catalogue of landraces of the area and conducted additional interviews to solve incongruences as stated in the methods section.

Second, although the classification of the local indicators of climate change impacts has been carried out according to the LICCI protocol (Reyes-García et al. 2020), there is necessarily a simplification of the observations perceived by the respondents in the act of classifying and clustering that may lead to a loss of part of the complexity of such perceptions. In a case study analyzing qualitative data, there is an inevitable reinterpretation of observations and a difficulty to establish cause-effect connections to reach conclusions (Queirós et al. 2017). For instance, interviewees might see an increase in birds as an increase in pests, and thus, this will be classified in the agricultural subsystem, even though bird increases could also be an impact in the terrestrial fauna subsystem.

Third, indicating exactly how climate change influences crop management and diversity can be complex, because the climatic system, although it is well defined in the LICCI protocol, is interrelated with the other systems (physical, biological, and socioeconomic). We deeply discuss this systemic interrelation in the subsequent section.

### Climate change impacts and adaptations in mountain home gardens

As several previous studies have pointed out, the perception of climate change impacts often drives the implementation of adaptation strategies in agricultural systems (Adger et al. 2009; Lereboullet et al. 2013; Abid et al. 2015). To reduce vulnerability and to strengthen resilience to environmental risk, it is important to understand perceptions of climate change risks (Vogel et al. 2007). In this study, respondents were asked how the weather had changed over the years. All the respondents (24 farmers) perceived some climate-related changes occurring in the area, so they believe that the climate is changing and that it is no longer as it was in the past.

Reports of changes in rainfall patterns, temperature, snowfall, agriculture, and transitions between seasons were the most abundant. These results are in line with other studies about climate change perceptions in regions with similar climatic conditions (Roco et al. 2015). These impacts affect the whole agroecosystem of Vall de Cardós and specifically the home gardens. Respondents mentioned that changes in the climatic system (precipitation, temperature, and seasons subsystems) affect the feasibility of rainfed crops and the irrigation of home gardens due to the decrease of useful rainfall. Changes in the climatic system also affect the productivity of nut trees, the frequency and types of crop pests and diseases, and the sowing and planting time. These climate change impacts are also well documented in the literature on climate change trends, specifically in Spain where they are expected to lead to an increased demand for irrigation, an increase in crop plagues, and a strong decrease in crop production are expected (Moreno et al. 2005; Vargas-Amelin and Pindado 2014). Moreover, changes in snow accumulation and snowfall have been detected previously in the Pyrenees (López-Moreno 2005).

The vulnerability of systems to climate change depends on the actual exposure to climate change, their sensitivity, and their adaptive capacity (IPCC 2001). Thus, climate change adaptation is not only limited by exogenous forces in terms of physical and ecological limits, but by societal factors. Indeed, perceived impacts of climate change in a local community could be an early indicator of potential adaptation measures to those changes (Adger et al. 2009; Abid et al. 2015).

Changes in crop management (understood as a form of adaptation) can largely reduce the potential impacts of climate change. This is why respondents were asked about changes in management practices in their home gardens (Reidsma et al. 2010). A clear adaptation found in this study is the fact that now they water or need to water more than before, which is related to the lower precipitation and the higher variability of rainfall patterns perceived by the respondents. However, the introduction of more water-demanding crop species such as vegetables and certain fruit trees is also influencing the increased water use in the area. In addition, interviewees observed that in the past they grew potato, pea, bean, and cereals in rainfed fields, but that now it would not be possible. The increase in irrigation to supplement rainwater and to compensate for the loss of water due to increased temperature is well documented in the literature (Deressa et al. 2009; Khanal et al. 2018).

Interestingly, home gardeners are now sowing or planting later than before. At first, this may seem inconsistent with the perceived temperature increase. However, this change is explained by an increase in climate variability and by the unpredictability of the frosts. Farmers stated that now it can freeze at any time, which in the past did not happen. Changing planting dates is an adaptation strategy used by home gardeners in different countries, such as Sri Lanka, India, or Ethiopia (Deressa et al. 2009; Marambe et al. 2012).

Nevertheless, the most mentioned management change is the increased use of pesticides, even if some home gardeners agreed that now people are more aware of the negative impacts of using them. Using more pesticides is also a common adaptation action taken by farmers, as reported in previous findings in other contexts (Khanal et al. 2018). This result also aligns with one of the most perceived climate change impacts in this case study, which is the increase of crop diseases and pests. Regarding this impact, the local perception of climate change captures the complexity of systemic effects. Several respondents pointed out the impact of the reduction of snowfall and freezing events in the insect life cycles, leading to an increase in the pests, which significantly affects some pest sensitive landraces, as also observed in apple orchards in Italy (Reedy et al. 2014). However, there is likely also a socio-economic explanation to this increase: the better access to agrochemical products farmers have experienced.

### Crop diversity in a changing climate and a globalized rural context

Home gardens in Vall de Cardós are a repository for the in situ conservation of crop genetic diversity as is the case with many home gardens studied in different regions in the world (Galluzzi et al. 2010; Calvet-Mir et al. 2011).

At the species level, eleven crop species have been abandoned mainly as a consequence of land-use changes. As mentioned above, the shifts towards a market-oriented economy and the mechanization and industrialization of agriculture led to the abandonment of most agricultural activities. Before, cereals and fodder crops used to be grown as there was a self-subsistence economy. Also, crops have been abandoned due to migration to urban areas in lowland zones, reducing the area under cultivation, as has been the case in other areas of the world (López-Moreno et al. 2008). Therefore, the loss of agrobiodiversity at the species level is mainly a consequence of socioeconomic and demographic changes, such as the rural exodus and the transformation of the traditional agrarian system that started in the 1960s in Spain (Aceituno-Mata 2010). Indeed, decisions taken in the rural context are mostly influenced by global socioeconomic trends (FAO 2004). However, our results highlight that climate change potentially intersects with socioeconomic drivers when we explain species' abandonment, as demonstrated in other areas in the world (Tripathi and Mishra 2017; Zhang et al. 2018). In Vall de Cardós, home gardeners stated that, given the rainfall decrease experienced nowadays, some species cannot be grown in rainfed lands as they used to grow in the past. Nevertheless, the changes in species’ composition show different trends in rainfed lands and home gardens. In our study, seven new species were reported in home gardens, while eleven were abandoned in rainfed plots. Therefore, there has been a loss of crop diversity at the species level in rainfed plots but not in home gardens systems, which was also reported in a previous study in another area in Spain (Aceituno-Mata 2010).

Crop species’ introduction in home gardens is also related both to a changing climate and a globalized rural context. On the one hand, people have incorporated new species due to improved access to new seeds through markets. Moreover, dietary changes have also caused an increase in the diversity of crop vegetable species, as it is a major driver of the introduction of species such as melon, cucumber, or pepper. This is in line with previous studies in home gardens of the Iberian Peninsula in which researchers found that before the 1960s, home gardens were focused on providing staple food, but nowadays dietary changes have decreased the number of staple crops and have increased the diversity of vegetable species cultivated in home gardens (Reyes-García et al. 2014). Although most respondents perceived this introduction of cultivated species as a consequence of entering the market economy, some home gardeners reported that it is also influenced by changes in climate, particularly by the warmer temperatures. This is the case of the eggplant, which has been grown for more than 30 years, but it is not very productive as it should be grown at lower altitudes and in warmer conditions. Similarly, the flat peach tree has not been traditionally cultivated in the valley, but currently, a farmer has this species since the temperature has increased in the last decades. Home gardeners also perceived that peppers (except *Capsicum annuum* var. *longum*) were not grown before because the climate was cooler and there was more humidity than nowadays. That is in line with the physiological characteristics of this crop species since pepper has a temperature range for germination higher than cool-season vegetables and low temperatures can reduce fruit quality (Abou-Hussein 2012). Indeed, crop shifts or the adoption of crops and crop varieties in response to new climate conditions is a process taking place in several places in the world, as described by other authors (Skarbø and VanderMolen 2016).

At the landraces level, a total of 59 local landraces were found, a number larger than in previous studies in similar valleys of the Catalan Pyrenees (Calvet-Mir et al. 2011; Riu-Bosoms et al. 2014). This can be explained by the presence of elder farmers that have lived in relatively high isolation and connectedness with nature. According to several authors, the key variables promoting crop diversity are market isolation, environmental heterogeneity, and generational effects, and the principal stewards of diversity are isolated and older farmers (Van Dusen and Taylor 2005).

Still, there is a loss in crop diversity associated with landrace abandonment (31 landraces have been abandoned or are in decline). The drivers of this loss are in line with the crop species changes mentioned before, including the increasing use of commercial seeds, the abandonment of cattle farming and rainfed crops, and the increase of crop pests and diseases. The overall diffusion of modern varieties has produced a decrease in the number of landraces and therefore an increase of the genetic erosion in agriculture, as reported in the literature for other parts of the world (Negri 2005; Barrera-Bassols et al. 2009). Landraces also require more intensive labor since home gardeners need to make seedbeds and some landraces are less productive than commercial varieties (Calvet-Mir et al. 2011).

Even so, people have expressed a preference for landraces rather than for commercial varieties and that is why they keep maintaining or have increased the cultivation of 28 landraces in home gardens, which is consistent with findings in previous studies in the high-mountain agroecosystems of the Iberian Peninsula, such as in Vall Fosca (Calvet-Mir et al. 2011). This preference is associated with the organoleptic properties of landraces (taste and color mostly), but also with their adaptability, as people stated that many commercial varieties are not adapted to the local environmental conditions of the area. Landrace’s resistance to cold and snow was also mentioned as a factor for their maintenance, although given the current climatic trends this might no longer be a favorable trait. These observations are in line with previous studies in other areas of the world that found that landraces’ genetic diversity is a defense against environmental changes (Negri 2005; Galluzzi et al. 2010).

## Conclusions

The results of this study suggest that both climate change impacts and socioeconomic factors are affecting home gardens in Vall de Cardós. Regarding climate, the main impacts perceived are changes in the rainfall, temperature, cryosphere, and seasons, which have cascading effects such as the imbalance of insects’ populations, the extended length of the summer cultivation season, and the increase of crop pests and diseases. Consequently, home gardeners are changing their management practices to cope with climate change through increasing irrigation, changing the sowing and planting date, and introducing new summer crop species that before were not viable. Although these adaptation strategies also respond to changes in the crop species that are now cultivated.

Crop diversity in the area is in decline, a change that is specifically affecting rainfed crop species. This decline is mostly driven by socioeconomic factors, but also by climate change. In fact, the agrobiodiversity loss found in this valley is mostly due to decreased profitability of agriculture, depopulation, high labor demand, and low productivity associated with some crop species and landraces in comparison to commercial varieties. Although climate change was not found as a primary driver of crop diversity loss, changes in the climatic system are affecting crop diversity. For instance, climatic changes are associated with the incorporation of new crop species from warmer climates or the abandonment of pest-sensitive landraces.

Still, even despite the overall crop diversity decline, home gardens in this valley are a repository of agrobiodiversity and are key for its in situ conservation since home gardeners have maintained the same crop species and still keep growing a large number of landraces. Landraces are preserved not only because of their organoleptic properties or their high resistance to cold and snow but also because of local appreciation and relation to culinary and plant use culture. This agrobiodiversity, if maintained, can play a key role in climate change adaptation, as landraces and traditional crop species grown in home gardens are better fitted to the environmental conditions of the valley. Therefore, the in situ conservation of local crop diversity and its associated traditional ecological knowledge is key to mitigate the increasing effects of climate change, especially in the Mediterranean area.

## Supplementary Information

ESM 1 (PDF 488 kb)

## Data Availability

Raw data in the form of audio recordings is available under request to protect respondent’s confidentiality.
