# Supplementary material for: Climate change in the Catalan Pyrenees intersects with socioeconomic factors to shape crop diversity and management
Source: Agron Sustain Dev. 2022 Sep 2;42(5):91. doi: 10.1007/s13593-022-00806-3 (PMC9438384; doi:10.1007/s13593-022-00806-3)
Supplement: Supplementary file 1 — (PDF 488 kb) [file 13593_2022_806_MOESM1_ESM.pdf]

**SUPPLEMENTARY MATERIALS**

# Climate change in the Catalan Pyrenees intersects with socioeconomic factors to shape crop diversity and management

Joana Blanch-Ramirez[1], Laura Calvet-Mir[1,2], Laura Aceituno-Mata[3], Petra Benyei[1*]

1 Institut de Ciència i Tecnologia Ambientals, Universitat Autònoma de Barcelona. Building Z Campus UAB, 08193 Bellaterra (Cerdanyola), Barcelona, Spain

2 Internet Interdisciplinary Institute (IN3), Universitat Oberta de Catalunya, Av. Carl Friedrich Gauss, 5. Parc Mediterrani de la Tecnologia, Castelldefels, 08860 Barcelona, Spain

3 Instituto Madrileño de Investigación y Desarrollo Rural, Agrario y Alimentario (IMIDRA), Finca El Encin, Autovía del Noreste A-2 Km 38,2. 28805 Alcalá de Henares, Spain

*Correspondence to petra.benyei@uab.cat

**METHODS**

## 1. Data collection guidelines

The LICCI data collection protocols (https://licci.eu/research-tools/) will be adapted to this specific research, which will be conducted in four sites with mountain agroecosystems: Alpujarra Granadina (Sierra Nevada), Vall de Cardòs (Pyrenees), Cabrales (Picos de Europa), and Sierra Norte (Sierra de Guadarrama). These sites have been selected because of their relative isolation (leading to better preserved traditional knowledge systems) and the fact that farmer communities in those sites have had long-term interactions with the environment.

Data will be collected using interviews and focus groups in 2-3 relatively homogeneous villages per site. For each site, 20-25 in-depth interviews will be conducted following two different sample strategies that will lead to two different sample groups.

Sample #1 (5-6 interviewees per site, 2-3 per village) will be selected by purposeful sampling, targeting older farmers that have had a continuous relationship with the local environment and have had experience with a diversity of agroecosystems in the area (e.g. pastures, homegardens, forestry-systems).

Sample #2 (15-20 interviewees per site, 5-7 per village) will be selected by quota sampling, targeting relatively elder farmers with a significant dedication to the homegarden agroecosystem (at least 25 years managing a homegarden). Age and gender quotas were deigned to include equal representation of male and female interviewees and equal representation of the following age groups: 60-70 years old, 70-80 years old, elder than 80 years old.

We will also conduct 2-3 focus groups (1 per village). Attendants to the focus groups will be sampled following purposeful sampling, targeting elder farmers with a continuous and significant dedication to agriculture, and specifically to home gardening.

If the villages in a given site have very few farmers, more than three villages can be sampled. Moreover, the sampled farmers can have other income-generating activities besides agriculture (animal husbandry, wage work, pensions etc.), but they should be acquainted sufficiently with the local cropping systems (have an agricultural past or have lived in the area long enough).

In the interviews to sample #1, we will ask open-ended questions focusing on:

1) The perceived local indicators of climate change impacts (LICCI) **in the site**

a. We will explore the **environmental changes the interviewee has noticed in the local ecosystem** with a focus on those affecting the agroecosystems. To obtain this information we will use a benchmark (e.g., since you were young) and ask specifically if they noticed changes in a) the seasons, temperature, rain, wind, storms (atmospheric system); b) the soil, water, terraces/land, snow (physical system), c) the crops, livestock, pastures, trees, birds, insects, diseases (biological system). This will be done keeping the LICCI tree in mind (https://licci.eu/ressources/licci-classes/index.html; see Reyes-García et al. 2020) and trying to encourage people to go to more precise levels of the tree and

directions (e.g., "increase/decrease in mean temperature" instead of "change in temperature"). We will avoid using the term climate change and ask about the moment in time when the change happened/was noticed in order to have a more specific timeline of events.

2) The cropping systems and crop management practices **in the village**

a. We will explore the different types of **cropping systems or cultivated fields** that were present in the village **and the management practices** that were used **in the homegardens**.

b. We will explore **how these cropping systems and practices have changed** through time.

c. We will explore if people link those **changes** to the previously reported LICCI (e.g., changes in soil, water, landscape, plants, insects, diseases…). With a **special focus on how homegarden management practices have changed to adapt to the local impacts of climate change.**

In the interviews to sample #2, we will ask open-ended questions focusing on:

3) The crop diversity (plant species and landraces) in the village's homegardens

a. We will list the **crop species and landraces grown in the past** (in the 50's-60's, before the green revolution) for food and income **in the village's homegardens.** This list will include the correspondence between the vernacular and Latin names of the botanical species, the ranking of crop species and landraces, and the origin of the landraces. Theses list can be done as well for one or two other very relevant agroecosystems for the people in the village (e.g., olive groves).

b. We will list the **crop species and landraces currently grown** for food and income **in the village's homegardens**. These lists can be done as well for one or two other very relevant agroecosystems for the people in the village (e.g., olive groves).

c. Based on a) and b), we will **list the trends in crop diversity** (changes from past to presently cultivated species and landraces) and explore if people link these changes to the previously reported LICCI (e.g., changes in soil, water, landscape, plants, insects, diseases…).

All these topics will be explored with the aid of a timeline (i.e., a piece of paper marking the relevant dates/events that everybody remembers) in order to better understand **since when the interviewees have noticed these changes**. In the timeline, we will mark some relevant events for the community and then try to mark the time when the interviewee started to notice the LICCI and the changes in crop diversity and management. We will also add to this timeline any significant event that affected the agroecosystems such as demographic or socio-economic changes that happened in the site. For more details on the interview guideline, see Appendix 1.

Each individual interview will add information on these topics until the sample goal is reached or information saturation is achieved (i.e., new interviewees do not add any new information). In the focus groups, the compiled information from the interviews (specifically the information that seems more controversial) will be confirmed and discussed, specifically focusing on:

1) **Perceived local indicators of climate change impacts on agroecosystems (LICCI).** We will bring the most relevant and controversial LICCI we find and ask if there is any other climate change impact that has not been mentioned. We will also bring the timeline to validate the aggregated information from the interviews.

2) **Changes/trends in homegarden crop species/landraces** over time. We will confirm the information on present and past crop species and landraces and the information from the aggregated timelines. We will also confirm the information about the causes/drivers of the changes noticed.

In case of continued sanitary emergency due to COVID-19 crisis (i.e., social distancing regulations), the focus groups will be substituted by a second round of confirmatory interviews, which can be done by telephone if needed.

The output of this data collection process will be:

1) A list of LICCI (looking specifically at LICCI affecting the agroecosystem elements).

2) A list of past and present cropping systems, management practices, crop species and landraces.

3) A list of crop diversity trends (changes comparing past and present crop species and landraces) and the drivers of these changes.

4) A timeline of how these events interrelate with each other and with other socio-economic and demographic changes in the area

These data will be processed and compiled using the LICCI app.

## 2. Interview guidelines (Spanish)

**Preguntas Muestra 1 (5-6 personas - purposeful sampling):**

Como decía, estamos mirando cosas que han cambiado en la agricultura y el medio natural de esta zona. Para entenderlo mejor, voy a usar una línea del tiempo (sacar papel continuo).

**1)** Primero, me gustaría saber si, en general, en el medio natural de la zona, ha percibido usted algún cambio respecto a cuando era joven (antes de casarse si estuvo casado/a, o en los años 50-60).

Por ejemplo, ha percibido algún cambio en las temperaturas, las lluvias, los vientos, las tormentas, las estaciones?

- Apuntar cambios en Tabla 1. IMPORTANTE: para cada cambio que mencionen, preguntar desde cuándo lo han notado. Usar línea del tiempo.

Y qué me dice de cambios en los suelos, es decir, en la tierra? O en los ríos y aguas subterráneas?

- Apuntar cambios en Tabla 1.

(si menciona algún cambio en los suelos y ríos, preguntar si están relacionados con los cambios mencionados anteriormente – temperatura, lluvias … - y apuntar esas causas en tabla 1)

Y en cuanto a las plantas y animales, ha cambiado algo? Por ejemplo, qué me dice de los pájaros, los animales salvajes o ganado? De los árboles? De las plantas silvestres y cultivos? De los insectos? Y las pestes? Han cambiado?

- Apuntar cambios en Tabla 1.

(si menciona algún cambio en los animales y plantas preguntar si están relacionados con los cambios mencionados anteriormente – temperatura, lluvias, suelos, rios…- y apuntar esas causas en Tabla 1).

Cuándo (más o menos) tuvieron lugar estos cambios que menciona? Hubo algún gran evento natural que usted recuerde y que quiera poner en la línea del tiempo?

-Apuntar en línea del tiempo

**2)** Y ahora más en concreto, me gustaría saber cómo ha cambiado la agricultura en este pueblo respecto a cuando usted era joven (antes de casarse si estuvo casado/a, o en los años 50-60).

Por ejemplo, me imagino que en la zona hay huertos familiares, pero, aparte, hay o hubo alguna otra actividad agrícola? Por ejemplo cultivo de arroz, garbanzos, maíz, trigo, espelta, girasoles, patatas, frutales, viñas, olivos…?

Y dónde se ponían los diferentes cultivos (en lo alto, en el valle, cerca del bosque, del río, de la casa etc.)? Eran las características del suelo diferentes en los diferentes campos? Se cultivaban cada año una vez o varias veces al año?

Y ha habido alguna actividad agraria especialmente relevante en el pasado que ya no exista? O que existiese un tiempo determinado pero se haya abandonado? Y alguna que haya empezado ahora o hace poco?

Y habéis cambiado el sitio donde se ponen los cultivos?

Cuándo pasaron estos cambios?

Recuerda algún otro cambio que considere importante en el paisaje o en la forma de vida de la zona?

-Apuntar en línea del tiempo y en tabla 3 del Anexo y guardar lista de campos de cultivos para preguntar más tarde por manejo y especies/variedades. Pero no entrar en especies y variedades en esta parte!

Y en general, qué tareas se realizaban en el huerto? Por ejemplo, cómo se regaba, abonaba, desherbaba, sembraba?

Y ahora se hace de otra manera? Han cambiado las tareas? Por ejemplo, se riega igual que antes? Los cultivos están en los mismo sitios? Se abona igual? Se siembra igual?

-Apuntar cambios en Tabla 2 y Tabla 3 del Anexo. Se pueden usar las categorías del LACCI: organización (tecnologías, marco de plantación, tiempos, ubicación) o actividades (abonado, siembra, desherbado, riego, cosecha) del huerto. Centrarlo en el huerto pero si sale algún otro cultivo se puede entrar en eso.

Y estos cambios, están relacionados con alguno de los cambios del medio natural que me ha comentado antes (por ejemplo, con los cambios en temperaturas, lluvias, ríos, suelos, insectos)? O fue por algún otro motivo?

-Apuntar factores en Tabla 2

IMPORTANTE preguntar por los elementos de la tabla 3 del anexo. Para cada agroecosistema nos piden:

-Location / topography: Describe where this type of field is usually located in the landscape, if relevant. Mention especially its location on the topographic gradient, proximity to water bodies, to households…

-Soil description (Indicate if this type of field presents particular soil characteristics (fertility, color, texture, humidity). You can indicate the general soil category it belongs to if you have this information…

- Main crops (List the main crop(s) cultivated in this type of field): NO ENTRAR MUY EN DETALLE

- Non-crop plants (Indicate if there are usually any other plants of agronomic interest that are not crop (e.g. shade trees))

- Number of cropping seasons in a year (Indicate how many cropping seasons are performed in the same plot during a year)

-------------- Muchas gracias por toda esta información, le importaría que le vuelva a visitar otro día para continuar la entrevista si fuese necesario? --------------------------------------------------------

**Preguntas Muestra 2 (15-20 quota sampling):**

Como decía, estamos mirando cosas que han cambiado en la agricultura y el medio natural de esta zona. Para entenderlo mejor, voy a usar una línea del tiempo (sacar papel continuo).

Hemos estado hablando con gente mayor de los pueblos y nos han contado que algunas cosas han cambiado en la temperatura, las lluvias, el agua, los suelos, las plantas y los animales (poner ejemplos concretos de la pregunta 1)

**3)** Ahora estamos intentando hacer una lista de plantas y variedades que se cultivan en este pueblo. Vamos a centrarnos en el huerto y en [el olivar] - uno/dos agroecosistemas muy relevantes que salgan de la lista de pregunta 2.

Cuando usted era joven (antes de casarse, en los años 50-60), qué plantas se cultivaban en el huerto (preguntar abierto primero)? Y que árboles?

- Apuntar lista en Tabla 3. Si hay datos previos, la tabla 3 deberá tener ya una lista y sólo se harán cruces en las que el informante mencione como actuales o pasadas. Si se olvidan de alguna, indagar más, pero es importante diferenciar lo que surgió espontáneamente y en el orden en que se mencionó para luego hacer indicadores de importancia y mención.

Y de las plantas que se cultivaban antes, eran todas iguales? O eran diferentes? Que variedades había (preguntar abierto)? Por ejemplo, qué tipo de [tomate, patata…] había?

- Ir planta por planta preguntando por variedades de esa planta. Apuntar lista en Tabla 3. Si hay datos previos, la tabla 3 deberá tener ya una lista y sólo se harán cruces en las que el informante mencione. Si se olvidan de alguna, indagar más, pero es importante diferenciar lo que surgió espontáneamente y en el orden en que se mencionó para luego hacer indicadores de importancia y mención.

Y ahora, esas plantas/árboles se siguen cultivando en el huerto? Y que plantas/arboles se cultivan ahora que antes no se cultivaban en el huerto?

- Ir planta por planta y apuntar lista en Tabla 3. Si hay datos previos, la tabla 3 deberá tener ya una lista y sólo se harán cruces en las que el informante mencione. Si se olvidan de alguna, indagar más. IMPORTANTE, si se han abandonado o introducido preguntar directamente cuándo y por qué (Pregunta 4)

Y ahora, esas variedades se siguen cultivando? Y que variedades o tipos de [tomate, patata…] se cultivan ahora que antes no se cultivaban?

- Apuntar lista en Tabla 3. Si hay datos previos, la tabla 3 deberá tener ya una lista y sólo se harán cruces en las que el informante mencione. Si se olvidan de alguna, indagar más, pero es importante diferenciar lo que surgió espontáneamente y en el orden en que se mencionó para luego hacer indicadores de importancia y mención.

IMPORTANTE preguntar por el origen de las variedades (ej., “y esta variedad que me dice, de dónde salieron las semillas? Se cultiva de siempre o son semillas comerciales o de vivero?”).

**4)** Entonces, por lo que hemos hablado, se ha dejado de cultivar:

-Usar Tabla 3 mirando las que se cultivaban antes y ya no.

Podría decirme por qué se han dejado de cultivar? Fue por alguno de los cambios del medio natural que le he comentado antes (por ejemplo, por los cambios en temperaturas, lluvias, ríos, suelos, insectos…)? O fue por algún otro motivo (su sabor, su olor, su tipo de manejo, su importancia cultural, su productividad etc.)?

-Usar lista de LICCI (Tabla 1) para ver si algunos de los cambios en temperatura, lluvias, suelos, ríos, plantas, o animales está relacionado aunque sea indirectamente con el abandono de especies o variedades de cultivo. IMPORTANTE, si es por cambio climático, preguntar qué característica tiene este cultivo que ha hecho que se abandone o deje de cultivar.

Y cuándo (más o menos) se abandonaron esos cultivos?

-Si se puede, poner en la línea del tiempo cuándo sucedieron los cambios aproximadamente

También, por lo que hemos hablado, se han mantenido o comenzado a cultivar:

-Usar Tabla 3 mirando las que se cultivan ahora y antes no.

Podría decirme por qué se han mantenido o comenzado a cultivar? Ha sido por alguno de los cambios del medio natural que me ha comentado antes (por ejemplo, por los cambios en temperaturas, lluvias, ríos, suelos, insectos)? O fue por algún otro motivo?

-Usar lista de LICCI (Tabla 1) para ver si algunos de los cambios en temperatura, lluvias, suelos, ríos, plantas, o animales está relacionado aunque sea indirectamente con la introducción de especies o

variedades de cultivo. IMPORTANTE, si es por cambio climático, preguntar qué característica tiene este cultivo que ha hecho que se haya mantenido o introducido.

Y cuándo (más o menos) se incorporaron esos cultivos?

-Si se puede, poner en la línea del tiempo cuándo sucedieron los cambios aproximadamente

Anotaciones:

**RESULTS**

**Table S3. List of changes in crop species cultivated**

| Species | Type of change | Drivers of change |
|---|---|---|
| *Triticum sp*<br>Blat (wheat) | Abandonment | Socio-economic changes: access to markets, migration, increased costs |
| *Cannabis sativa ssp. Sativa*<br>Cànem (hemp) | Abandonment | Abandonment of cattle farming and<br>Socio-economic changes: access to markets, migration, increased costs |
| *Cicer arietinum*<br>Cigrons (chickpeas) | Abandonment | Socio-economic changes: access to markets, migration, increased costs |
| *Avena sativa*<br>Civada (oat) | Abandonment | Socio-economic changes: access to markets, migration, increased costs |
| *Helianthus annuus*<br>Gira-sols (sunflower) | Abandonment | Abandonment of cattle farming |
| *Lens culinaris*<br>Llenties (lentils) | Abandonment | Socio-economic changes: access to markets, migration, increased costs |
| *Hordeum vulgare*<br>Ordi (barley) | Abandonment | Socio-economic changes: access to markets, migration, increased costs |
| *Zea mays*<br>Panís (corn) | Abandonment | Socio-economic changes: access to markets, migration, increased costs |
| *Cynara cardunculus*<br>Herbacol (thistle) | Abandonment | Abandonment of cow dairy farming |
| *Beta vulgaris var. Conditiva*<br>Remolatxa blanca (white beet) | Abandonment | Abandonment of cattle farming |

| | | |
|---|---|---|
| *Secale cereale*<br>Sègol (rye) | Abandonment | Socio-economic changes: access to markets, migration, increased costs |
| *Solanum melongena*<br>Albergínia (eggplant) | Introduction | Increase in temperature, dietary changes, access to commercial seed |
| *Cucumis sativus*<br>Cogombre (cucumber) | Introduction | Increase in temperature, dietary changes, access to commercial seed |
| *Cucumis melo*<br>Meló (melon) | Introduction | Increase in temperature, dietary changes, access to commercial seed |
| *Prunus persica var. Platycarpa*<br>Préssec pla (flat peach) | Introduction | Increase in temperature, dietary changes, access to commercial seed |
| *Citrullus lanatus*<br>Síndria (watermelon) | Introduction | Increase in temperature, dietary changes, access to commercial seed |
| *Capsicum*<br>Pebrot (pepper) | Introduction | Increase in temperature, dietary changes, access to commercial seed |
| *Beta vulgaris var. Conditiva*<br>Remolatxa vermella (red beet) | Introduction | Dietary changes, access to commercial seed |
| *Pisum sativum*<br>Pèsol (peas) | Changed from rainfed land to home gardens / In decline | Socio-economic changes, changes in rainfall patterns |
| *Vicia faba*<br>Fava (fava beans) | Changed from rainfed land to home gardens / In decline | Socio-economic changes, changes in rainfall patterns |
| *Solanum tuberosum*<br>Patata (potatoes) | Changed from rainfed land to home gardens / In decline | Abandonment of cattle farming, changes in rainfall patterns |

**Table S4.** Changes in traditional landraces grown in home gardens in the study area.

| Species Latin name | Landrace name | Presence before Yes | Presence now Yes | Type of change | Drivers of change and motivations for maintenance / abandonment | Climatic Factor |
|---|---|---|---|---|---|---|
| *Allium sativum* L. | All del país | 1 | 0 | Abandonment | Use of commercial cloves | No |
| *Apium graveolens* L. | Àpit del país | 4 | 0 | Abandonment | Use of commercial seed/seedlings | No |
| *Corylus avellana L.* | Avellaner bord | 2 | 2 | No change | | |
| *Beta vulgaris* var. *cicla* (L.) K.Koch | Bleda de sempre, de tocino, verda | 7 | 5 | Presence decrease | Valued for its resistance to cold and not valued for its taste | No |
| *Cucurbita ficifolia* C.D. Bouché | Carbassa de cabell d'àngel | 2 | 2 | No change | Valued for making jam | No |
| *Cucurbita maxima* Duchesne | Carbassa groga | 4 | 4 | No change | | No |
| *Cucurbita moschata* | Carbassa de pera | 1 | 1 | No change | | |
| *Cucurbita maxima* Duchesne | Carbassa de rabequet grossa | 9 | 5 | Presence decrease | Not valued due to its big size and bland taste / Abandonment of cattle farming and rainfed crops | Yes |
| *Cucurbita pepo* L. | Carbassó blanc | 1 | 1 | No change | | No |
| *Allium cepa* L. | Ceba valenciana, de sempre, de guardar | 16 | 15 | Presence decrease | Valued for its long conservation / Abandonment of rainfed crops | Yes |
| *Prunus avium* L. | Cirerer salranes | 5 | 4 | Presence decrease | Not valued due to its pest vulnerability | Yes |
| *Cydonia oblonga* Mill. | Codonyer del país | 2 | 2 | No change | Valued for their taste | No |
| *Brassica oleracea* L. | Col de tocino, comuna o de ruc | 19 | 19 | Abundance decrease | Valued for its frost resistance and quality / Abandonment of cattle farming and rainfed crops | Yes |
| *Brassica oleracea* L. | Col de cabdell | 6 | 6 | No change | Valued for its taste | No |
| *Brassica oleracea* L. | Col borrugada | 4 | 4 | No change | Valued for its taste | No |
| *Brassica oleracea* L. | Col de cor de bou, de paperina | 3 | 4 | Presence increase / Abundance decrease | | |
| *Brassica oleracea* L. | Col de cabdell plana | 2 | 2 | No change | Valued for its cold resistance and its use in winter salads | No |
| *Lactuca sativa* L. | Enciam de cassoleta | 5 | 3 | Presence increase | Use of commercial seedlings | No |
| *Lactuca sativa* L. | Enciam de cassoleta blanc | 1 | 0 | Abandonment | Use of commercial seedlings | No |

| | | | | | | |
|---|---|---|---|---|---|---|
| *Lactuca sativa* L. | Enciam de tres ulls | 4 | 4 | Abundance decrease | Valued for its cold resistance / Not valued because its short harvest period | Yes |
| *Lactuca sativa* L. | Enciam escaroler | 1 | 1 | No change | Valued for its cold resistance | No |
| *Lactuca sativa* L. | Enciam negre, d'hivern | 13 | 13 | Abundance decrease | Valued for its cold and frost resistance/ Not valued because now it has a shorter harvest period due to warmer conditions | Yes |
| *Cichorium endivia* var. *crispum* Lam. | Escarola arrissada | 3 | 1 | Presence decrease | Not valued because it costs a lot to clean it and slugs are a problem for this plant | No |
| *Cichorium endivia* var. *crispum* Lam | Escarola de cabell d'àngel | 2 | 2 | No change | Valued for its very curly and white leaves | No |
| *Cichorium endivia* var. *crispum* Lam | Escarola de fulla ampla | 3 | 2 | Presence decrease | | No |
| *Vicia faba* L. | Faves favolins | 1 | 1 | No change | Valued for its very tender fruits | No |
| *Spinacia oleracea* L. | Espinacs de sempre | 2 | 2 | No change | Valued for its long harvest period | No |
| *Fragaria x ananassa (Weston) Duchesne ex Rozier* | Maduixera de sempre | 2 | 2 | No change | Valued for its sweet taste | No |
| *Fragaria* sp. | Maduixera de Civís | 1 | 0 | Abandonment | Not valued because it cannot be transported | No |
| *Phaseolus vulgaris* L. | Mongeta avellaneta | 1 | 1 | No change | Difficulty to find seeds | No |
| *Phaseolus vulgaris* L. | Mongeta blanca, de granar | 5 | 3 | Presence decrease | Abandonment of grain production / Abandonment of rainfed crops | Yes |
| *Phaseolus vulgaris* L. | Mongeta del ganxet | 2 | 1 | Presence decrease | Use of commercial seedlings | No |
| *Phaseolus vulgaris* L. | Mongeta de mata baixa groga, tavellot | 2 | 1 | Presence decrease | Colour and taste not valued | No |
| *Phaseolus vulgaris* L. | Mongeta negra i marró | 2 | 2 | Abundance decrease | Difficulty to find seeds | No |
| *Solanum tuberosum* L. | Patata quarantena | 2 | 1 | Presence decrease | Abandonment of rainfed crops | Yes |
| *Solanum tuberosum* L. | Patata del bufet | 1 | 1 | Abundance decrease | Valued for their quality but difficult to peel | No |
| *Solanum tuberosum* L. | Patata de Burgos | 2 | 1 | Presence decrease | Valued for its reproductive capacity but harsh | No |
| *Capsicum annuum* L. | Pebrot bitxo | 7 | 8 | Presence increase | Because there are now warmer conditions | Yes |
| *Pyrus communis* L. | Perera de Cardós, d'olla | 12 | 11 | Presence decrease | Valued for its taste but very pest and diseases sensitive | Yes |

| | | | | | | |
|---|---|---|---|---|---|---|
| *Pyrus communis* L. | Perera "d'aigua" | 1 | 1 | No change | | No |
| *Pisum sativum* L. subsp. *sativum* | Pèsol de mata baixa | 2 | 1 | Presence decrease | | No |
| *Pisum sativum* L. subsp. *sativum* | Pèsol de mata alta, d’emparrar | 2 | 1 | Presence decrease | | No |
| *Pisum sativum* L. subsp. *sativum* | Pèsol tirabec | 1 | 1 | No change | | No |
| *Malus domestica* L. | Pomera morro de llebre | 2 | 2 | Abundance decrease | Valued for its long conservation but taste not valued | No |
| *Malus domestica* L. | Pomera del país, de Cardós | 5 | 4 | Presence decrease | Valued for its long conservation but sensitive to pests | Yes |
| *Malus domestica* L. | Pomera del cor gelat | 1 | 1 | No change | | No |
| *Prunus persica* (L.) Batsch | Presseguer de vinya | 1 | 1 | No change | | No |
| *Prunus persica* (L.) Batsch | Presseguer blanc | 4 | 4 | Abundance decrease | Not valued because it cannot be transported, and it is very pest sensitive | Yes |
| *Prunus domestica* subsp. *insititia* (L.) C.K.Schneid. | Prinyoners cascabellicos | 3 | 3 | No change | Valued for cooking | No |
| *Prunus domestica* subsp. *insititia* (L.) C.K.Schneid. | Prinyoners Peters | 1 | 1 | No change | Valued for its sweet taste | No |
| *Prunus domestica* subsp. *insititia* (L.) C.K.Schneid. | Prinyoners | 9 | 8 | Presence decrease | | No |
| *Prunus domestica L.* | Prunera morada | 1 | 1 | No change | | No |
| *Prunus domestica L.* | Prunera clàudia | 1 | 1 | No change | | No |
| *Prunus domestica L.* | Prunera allargada, “de llàgrima” | 2 | 2 | No change | | No |
| *Beta vulgaris* L. var. *rapacea* W. D. J. Koch | Remolatxa blanca, dels porcs | 4 | 0 | Abandonment | Abandonment of cattle farming | No |
| *Solanum lycopersicum* L. | Tomàquet del país, de sempre, de casa | 14 | 4 | Presence decrease | Valued for its taste / Use of commercial seed | No |
| *Solanum lycopersicum* L. | Tomàquet rosa | 3 | 6 | Presence increase | Valued for its sweet taste | No |
| *Solanum lycopersicum* L. | Tomàquet de pera, de popa de vaca | 6 | 12 | Presence increase | Valued for its taste | No |
| *Solanum lycopersicum* L. | Tomàquet quarantena | 2 | 0 | Aba | | No |
